# Systematic Characterization of Novel Immune Gene Signatures Predicts Prognostic Factors in Hepatocellular Carcinoma

**DOI:** 10.3389/fcell.2021.686664

**Published:** 2021-09-23

**Authors:** Dafeng Xu, Yu Wang, Jincai Wu, Yuliang Zhang, Zhehao Liu, Yonghai Chen, Jinfang Zheng

**Affiliations:** ^1^Department of Hepatobiliary and Pancreatic Surgery, Hainan General Hospital, Hainan Affiliated Hospital of Hainan Medical University, Haikou, China; ^2^Geriatric Medicine Center, Hainan General Hospital, Hainan Affiliated Hospital of Hainan Medical University, Haikou, China; ^3^Department of Otolaryngology-Head and Neck Surgery, Hainan General Hospital, Hainan Affiliated Hospital of Hainan Medical University, Haikou, China

**Keywords:** immune gene signatures, HCC, prognosis, tumor immune infiltration cell, pan-cancer

## Abstract

**Background:** The prognosis of patients with hepatocellular carcinoma (HCC) is negatively affected by the lack of effective prognostic indicators. The change of tumor immune microenvironment promotes the development of HCC. This study explored new markers and predicted the prognosis of HCC patients by systematically analyzing immune characteristic genes.

**Methods:** Immune-related genes were obtained, and the differentially expressed immune genes (DEIGs) between tumor and para-cancer samples were identified and analyzed using gene expression profiles from TCGA, HCCDB, and GEO databases. An immune prognosis model was also constructed to evaluate the predictive performance in different cohorts. The high and low groups were divided based on the risk score of the model, and different algorithms were used to evaluate the tumor immune infiltration cell (TIIC). The expression and prognosis of core genes in pan-cancer cohorts were analyzed, and gene enrichment analysis was performed using clusterProfiler. Finally, the expression of the hub genes of the model was validated by clinical samples.

**Results:** Based on the analysis of 730 immune-related genes, we identified 64 common DEIGs. These genes were enriched in the tumor immunologic related signaling pathways. The first 15 genes were selected using RankAggreg analysis, and all the genes showed a consistent expression trend across multi-cohorts. Based on lasso cox regression analysis, a 5-gene signature risk model (ATG10, IL18RAP, PRKCD, SLC11A1, and SPP1) was constructed. The signature has strong robustness and can stabilize different cohorts (TCGA-LIHC, HCCDB18, and GSE14520). Compared with other existing models, our model has better performance. CIBERSORT was used to assess the landscape maps of 22 types of immune cells in TCGA, GSE14520, and HCCDB18 cohorts, and found a consistent trend in the distribution of TIIC. In the high-risk score group, scores of Macrophages M1, Mast cell resting, and T cells CD8 were significantly lower than those of the low-risk score group. Different immune expression characteristics, lead to the different prognosis. Western blot demonstrated that ATG10, PRKCD, and SPP1 were highly expressed in cancer tissues, while IL18RAP and SLC11A1 expression in cancer tissues was lower. In addition, IL18RAP has a highly positive correlation with B cell, macrophage, Neutrophil, Dendritic cell, CD8 cell, and CD4 cell. The SPP1, PRKCD, and SLC11A1 genes have the strongest correlation with macrophages. The expression of ATG10, IL18RAP, PRKCD, SLC11A1, and SPP1 genes varies among different immune subtypes and between different T stages.

**Conclusion:** The 5-immu-gene signature constructed in this study could be utilized as a new prognostic marker for patients with HCC.

## Background

Hepatocellular Carcinoma (HCC) is the most common type of liver cancer and one of the leading causes of cancer death worldwide. In 2020, there were an estimated 42,810 new liver cancer cases and 30,160 related deaths in the United States (Siegel et al., 2020). The major risk factors for liver cancer include chronic Hepatitis B virus infection, hepatitis C infection, obesity, and alcoholism (El-Serag and Rudolph, 2007). At present, the treatment of HCC mainly includes surgical resection, transvascular chemoembolization, radiofrequency ablation, liver transplantation, molecular targeted therapy, and systemic chemotherapy. However, due to the concealed onset of HCC, more than 80% of patients are diagnosed at an advanced stage. The above treatment methods are usually insufficient, and the 5-year overall survival rate is less than 10% (Forner et al., 2018). Therefore, new therapies with different mechanisms are urgently needed to improve the prognosis of HCC patients. Immunotherapy has gradually become another milestone after traditional radiotherapy and chemotherapy.

The liver is a special immune tolerance organ which can effectively escape the immune response. Immunotherapy can enhance the immune response, stimulate tumor-specific immunity, break immune tolerance, and reactivate immune cells, for the purpose of recognizing and destroying tumor cells. In patients with Liver Hepatocellular Carcinoma (LIHC), CTLA-4 is often overexpressed in the liver’s dendritic cells, which inhibits T cell activation and proliferation and reduces its ability to recognize tumor antigens (Sangro et al., 2013). PD-1 can inhibit the activation of antigen-specific T cells by binding with its ligand PD-L1, which can decrease the immune response of tumor microenvironment to T cells and lead to immune escape (Yarchoan et al., 2019). Anti-PD-1/PD-L1 nivolumab and pembrolizumab are currently used in the treatment of HCC. Although immunotherapy has made progress in patients with liver cancer, the response rate to immunotherapy varies from patient to patient, even regarding drug resistance. Therefore, sensitive and specific predictive biomarkers must be identified to maximize the efficacy of immunotherapy. It is necessary to determine the role of tumor immune microenvironment and immune-related genes in the diagnosis and prognosis of HCC.

In recent years, the rapid development of high-throughput sequencing technology has caused the rapid emergence of retrospective studies using public databases to provide new guidance for prognosis prediction and clinical treatment of tumors. An immune-related 7-microRNA signature was constructed to predict the prognosis of patients with HCC (Li et al., 2021). Zhang et al. (2020) used an immune-associated lncRNA signature to predict the efficacy of immune checkpoint therapy in patients with HCC. Zhao et al. (2020) constructed a 4-gene signature using immune checkpoint-related to guide the diagnosis and prognosis of HCC patients. A 5-gene signature constructed by Tian et al. (2019) using tissue-associated immune biomarkers can be used to predict the survival outcome of patients with early/middle stage HCC. In this study, we systematically described the immune characteristics and immunophenotypes of HCC tumor microenvironment using 730 immune-related genes based on four data sets. 64 immune-related genes were identified. These genes were closely related to the immune pathways including NF–kappa B signaling pathway, TNF signaling pathway, and Cytokine receptor interaction. A 5-gene signature was constructed to predict the prognosis of patients with HCC. The results of this study highlight how tumor microenvironment promotes the prognosis of patients with HCC, and can explain the complexity of immune microenvironment of HCC and provide new ideas regarding immunotherapy for HCC patients.

## Materials and Methods

### Data Source and Preprocessing

RNA-Seq data and clinical follow-up data of LIHC data set and HCCDB18 data set were downloaded from the TCGA database and the HCCDB database^1^, respectively. GSE14520 data set with survival data, in addition to GSE22058, GSE25097, GSE64041, and GSE36376 data sets containing tumor and para-cancerous samples without survival data, were downloaded from the Gene Expression Omnibus (GEO) database.

The RNA-seq data of TCGA-LIHC were processed in the following steps: (1) removing samples with no clinical follow-up information; (2) removing samples with no survival time; (3) removing samples with no Status; (4) transforming ensemble into gene symbols; and (5) taking the median with multiple gene symbol expressions. GEO data sets were processed in the following steps: (1) removing samples without clinical follow-up information; (2) removing samples without survival time and survival status; (3) converting probes into gene symbols; (4) removing the probe when corresponding to multiple genes; and (5) taking the median value of the expression of multiple gene symbols. The RNA-seq data of HCCDB18 were processed in the following steps: (1) removing samples without clinical follow-up information; (2) removing samples without survival time; (3) removing samples without status; and (4) removing samples without expression spectrum data.

After data-preprocessing, there were 197 samples, including 100 tumor samples and 97 para-cancerous samples, in GSE22058; 511 samples, including 268 tumor samples and 243 para-cancerous samples, in GSE25097; 120 samples, including 60 tumor samples and 60 para-cancerous samples, in GSE64041; and 433 samples, including 240 tumor samples and 193 para-cancerous samples, in GSE36376. The samples clinical statistics were shown in Table 1.

**TABLE 1:** Clinical statistics of the sample.

Data	Tumor	Adjacent	Total
GSE22058	100	97	197
GSE25097	268	243	511
GSE64041	60	60	120
GSE36376	240	193	433
GSE14520	225	220	445
HCCDB18	212	177	389
TCGA	365	50	415
			2095

There were 365 samples for TCGA-LIHC, 203 samples for HCCDB18, and 221 samples for GSE14520 with survival data after pre-processing. Clinical statistics of the sample are shown in Table 2.

**TABLE 2:** Samples with survival information.

Clinical features	TCGA-LIHC	HCCDB18	GSE14520
**OS**			
0	235	168	136
1	130	35	85
**T stage**			
T1	180		
T2	91		
T3	78		
T4	13		
TX	3		
**N stage**			
N0	248		
N1	4		
NX	113		
**M stage**			
M0	263		
M1	3		
MX	99		
**Stage**			
I	170		
II	84		
III	83		
IV	4		
X	24		
Grade			
G1	55		
G2	175		
G3	118		
G4	12		
GX	5		
**Gender**			
Male	246		
Female	119		
**Age**			
≤60	173		
>60	192		
**Recurrence**			
YES	167		
NO	198		

### Identification of Differentially Expressed Genes (DEGs) and Analysis of KEGG Pathway and GO Enrichment

The limma package (Ritchie et al., 2015) was used to analyze the differentially expressed immune genes of 730 immune genes in tumor samples and para-cancerous samples from four data sets, and filter with False discovery rate (FDR) < 0.05 and | FC | > 1.2 as the threshold. The differentially expressed immune genes were analyzed by using the R package WebGestaltR (v0.4.2) for Kyoto Encyclopedia of Genes and Genomes (KEGG) pathway analysis and Gene ontology (GO) function enrichment analysis. Rankaggreg was used to identify genes with the most significant variation in the differentially expressed immune genes.

### Random Grouping of Training Set Samples

The 365 samples in the TCGA data set were divided into a training set and a validation set. In order to prevent random assignment bias from compromising the stability of subsequent modeling, all samples were placed in random grouping 100 times in advance. The grouping sampling was performed according to the ratio of training set: validation set = 1:1. The most suitable training sets and validation sets were selected according to the following conditions: (1) the two groups were similar in age distribution, sex, follow-up time, and patient mortality ratio; (2) the sample size of the two groups was close after randomly clustering the gene expression profiling data sets. There were 182 samples in the final training set and 183 samples in the validation set. The training set and validation set of the TCGA data are shown in Table 3. The training set and validation set were tested by chi-square test. The result showed that our grouping is reasonable.

**TABLE 3:** TCGA training set and validation set sample information.

Clinical features	TCGA-LIHC train	TCGA-LIHC test
**OS**		
0	122	113
1	60	70
**T stage**		
T1	93	87
T2	47	44
T3	32	46
T4	7	6
TX	3	0
**N stage**		
N0	114	134
N1	0	4
NX	68	45
**M stage**		
M0	122	141
M1	2	1
MX	58	41
**Stage**		
I	87	83
II	42	42
III	33	50
IV	2	2
X	18	6
**Grade**		
G1	29	26
G2	89	86
G3	54	64
G4	6	6
GX	4	1
**Gender**		
Male	121	125
Female	61	58
Age		
≤60	91	82
>60	91	101
**Recurrence**		
YES	76	91
NO	106	92

### Univariate Cox Regression and Lasso Regression Analysis

R package survival coxph function was used to conduct univariate Cox proportional hazard regression analysis based on the survival data of each immune gene in the training set data. *P* < 0.05 was selected as the threshold for filtering. Lasso regression (Kukreja et al., 2006) was further compressed using the R package glmnet to reduce the number of genes in the risk model. Lasso regression is a kind of compression estimation. A more refined model is obtained by constructing a penalty function, which compresses some coefficients and sets some to zero. This retains the advantage of subset contraction, which is a kind of biased estimation with complex collinear data. Consequently, one can realize the selection of variables as well as parameter estimation and better solve the problem of multicollinearity in regression analysis. The Akaike information criterion (AIC) was further used to reduce the number of model genes. The STEPAIC method in the MASS package starts from the most complex model and removes one variable, to reduce the AIC. The smaller the value, the better the model is, as it demonstrates that the model can obtain enough fitting degree with fewer parameters.

### Expression and Prognosis Value of the Five Genes in Pan-Cancer

The transcriptome data of 33 cancers and the normal tissue data were downloaded from the UCSC Xena database and the GTEX database, respectively. The box maps of ATG10, IL18RAP, PRKCD, SLC11A1, and SPP1 in tumor tissues and normal tissues of 33 cancer types were plotted using the ggplot2 package. The prognosis forest map of each gene in 33 cancer types was also drawn with the ggplot2 package.

### Sample Collection and Western Blotting

Liver hepatocellular carcinoma and adjacent normal tissues were collected from 4 patients, immediately placed in liquid nitrogen, and preserved at −80°C. Take the tumor tissue and normal tissue adjacent to the cancer into small pieces and put them into the tube, add lysis buffer RIPA (1% Triton X-100, 50 mM Tris-HCl pH7.4, 150 mM Na Cl, 10 mM EDTA, 100 mM Na F, 1 mM Na 3 VO 4, 1 mM PMSF, 2 μg/ml Aprotinin) (1 ml lysate is added to 250mg tissue). Use a homogenizer to homogenize at low speed for 30 s each time, and ice bath for 1 min between each time until the tissue is completely lysed. Centrifuge at 13,000 rpm for 25 min, take the supernatant, and quantify the protein by Coomassie brilliant blue method. After mixing with 3 × sample buffer, boil for 5 min. The sample (30–50 μg/lane) was electrophoresed in a 12% SDS-polypropylene gel for 3 h, and then transferred to a nitrocellulose membrane (voltage: 2 mV/cm^2^; time: 120 min). After sealing with 5% skimmed milk for 1 h, cut the transfer film according to the molecular weight marked by the pre-stained Marker, and add the primary antibodies separately at 4°C overnight. After washing 4 times with TTBS, add secondary antibody (1:2000) for 30 min at room temperature. After washing 4 times with TTBS again, the color will be developed by ECL method. The primary antibodies are as follows: ATG10 (1:1000, ab124711, abcam), PRKCD (1:1000, SAB4300539, sigma), IL18RAP (1:1000, AV42154, sigma), SLC11A1 (1:10000, SAB2108019, sigma), SPP1 (1:1000, ab214050, Sigma).

After rinsing 3 times (10 min each time) with tris-buffered saline, the membrane was incubated with horseradish peroxidase-conjugated secondary antibody against rabbit IgG (1:5000, Amersham Bioscience, Piscataway, NJ, United States) for 1 h at room temperature. After washout, the membrane was developed using enhanced chemiluminescence reagents (Pierce, Rockford, IL, United States) and visualized using a chemiluminescence system (PTC-200, Bio-Rad Laboratories, Hercules, CA, United States). All Western blots were repeated 3 times.

### Immunologic Correlation of the Five Genes in Pan-Cancer

The immune score and matrix score of each patient were assessed using the estimate package, while the levels of ATG10, IL18RAP, PRKCD, SLC11A1, and SPP1 in pan-cancer were analyzed. The corrplot package was used to visualize the correlation between each gene and the immune score and matrix score of each cancer type. A timer database^2^ was used to analyze the association of five genes with immune cells.

### Clinical Correlation and Pathway Enrichment of the Five Genes in HCC

The levels of the five genes in different clinical stages, tumor grade, T stage, N stage, and M stage in HCC was further analyzed. The patients with HCC were divided into high- and low-risk groups according to levels of the five genes, and the single gene Gene Set Enrichment Analysis (GSEA) of the five genes was analyzed using the clusterProfiler package.

## Results

### 64 Differentially Expressed Immune Genes Were Identified

**FIGURE 1:**
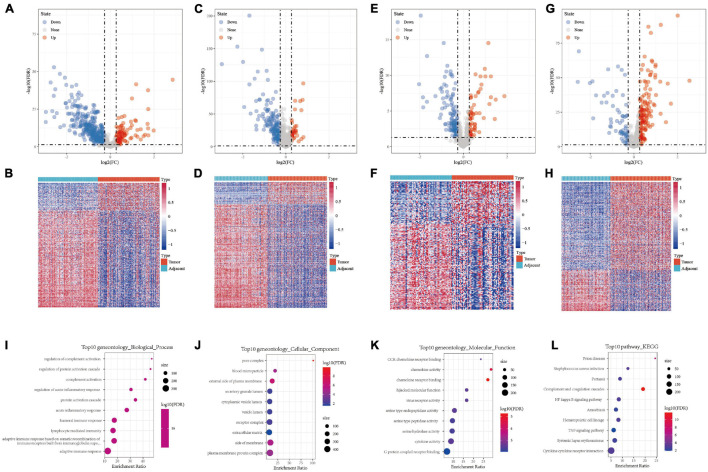
**(A,B)** Volcano map and heat map of differentially expressed genes in GSE22058. **(C,D)** Volcano map and heat map of differentially expressed genes in GSE25097. **(E,F)** Volcano map and heat map of differentially expressed genes in GSE64041. **(G,H)** Volcano map and heat map of differentially expressed genes in GSE36376. **(I)** Bubble chart of differentially expressed genes in biological process. **(J)** Bubble chart of differentially expressed genes in cellular component. **(K)** Bubble chart of differentially expressed genes in molecular function. **(L)** Bubble chart of differentially expressed genes in KEGG pathway.

A total of 443, 201, 184, and 205 differentially expressed immune genes were identified in the GSE22058, GSE25097, GSE64041, and GSE36376 data sets, respectively. The differentially expressed immune genes were mapped by volcanoes and heatmaps (Figures 1A–H, S2-S5.CSV). A total of 64 differentially expressed immune genes were obtained from the intersection of differentially expressed immune genes in four data sets (Supplementary Figure 1A). The results of GO functional enrichment analysis demonstrate that there were 277 items with significant differences in BP, the top ten of which are shown in Figure 1I. There were 15 items with significant differences in CC, the top ten of which are shown in Figure 1J. There were 16 items with significant differences in MF, the top 16 of which are shown in Figure 1K. The results of the first ten KEGG pathways showed that the gene was significantly enriched in the NF–kappa B signaling pathway, TNF signaling pathway, and Cytokine receptor interaction (Figure 1L).

### The Expression of Differentially Expressed Immune Genes in HCC Differed From That in Normal Tissues

**FIGURE 2:**
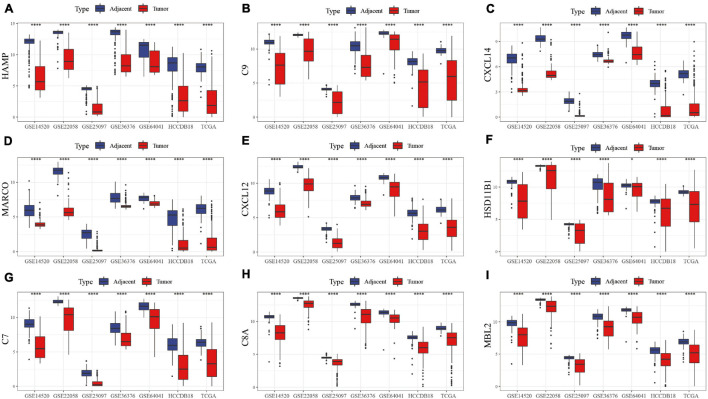
Box map of DEGs expression in tumor and para-cancer samples in different data sets. The differences in the expression of **(A)** HAMP, **(B)** C9, **(C)** CXCL14, **(D)** MARCO, **(E)** CXCL12, **(F)** HSD11B1, **(G)** C7, **(H)** C8A, and **(I)** MBL2 genes, respectively, in tumor and adjacent samples.

The first 15 genes with the most significant differentially expressed genes were identified including HAMP, C9, CXCL14, MARCO, CXCL12, HSD11B1, C7, C8A, MBL2, C6, EGR1, CFP, C8B, CXCL2, and CCL19. GSE22058, GSE25097, GSE64041, GSE36376, TCGA, HCCDB18, and GSE14520 were used to analyze the expression of these genes in tumor samples and para-cancer samples. HAMP, C9, CXCL14, MARCO, CXCL12, HSD11B1, C7, C8A, and MBL2 were differentially expressed, as shown in Figure 2. The differentially expressed profile of the other genes are shown in Supplementary Figure 1B. The results demonstrated significant differences in the expression of these hub genes between HCC and adjacent tissues, suggesting that immune-related genes play an important role in HCC.

### The Construction of Risk Model

**FIGURE 3:**
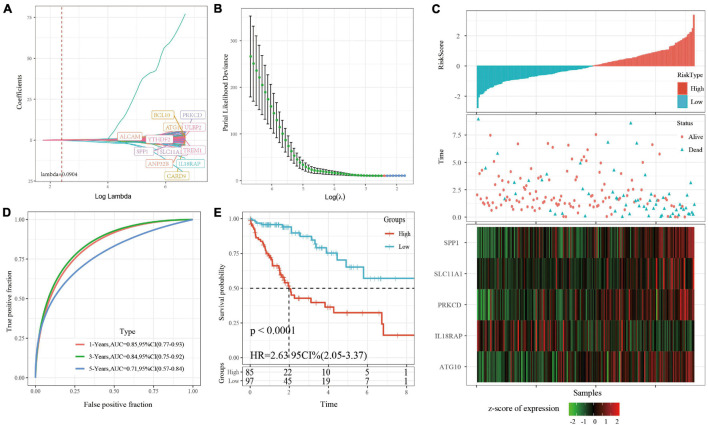
**(A)** For the changing trajectory of each independent variable, the horizontal axis represents the log value of the independent variable lambda, and the vertical axis represents the coefficient of the independent variable. **(B)** The confidence interval under each lambda. **(C)** RiskScore, survival time, survival status, and 5-gene expression in TCGA training set. **(D)** ROC curve and AUC of 5-gene signature classification. **(E)** The KM survival curve distribution of 5-gene signature in training set.

A univariate cox regression model was used to analyze 730 immune-related genes, which produced 132 DEGs, as shown in S8.csv. Lasso regression was used to further compress the genes. The change track of each independent variable is shown in Figure 3A, in which we can see that with the gradual increase of lambda, the number of independent variable coefficients gradually trends to 0. We used fivefold cross-validation to build the model and analyzed the confidence interval under each lambda, as shown in Figure 3B. The results showed that the model is optimal when lambda = 0. 0904, and the 12 genes were obtained when lambda = 0. 0904. The AIC regression further compressed the number of the hub genes into five: ATG10, IL18RAP, PRKCD, SLC11A1, and SPP1. The KM curve of the five genes is shown in Supplementary Figure 2, which shows that all the five genes could separate TCGA training set samples significantly (*p* < 0.05). The 5-gene signature formula is as follows: RiskScore = 0.841 ^∗^ ATG10-0.989 ^∗^ IL18RAP + 0.507 ^∗^ PRKCD + 0.437 ^∗^ SLC11A1 + 0.104 ^∗^ SPP1.

RiskScore was calculated for each sample based on level of expression. The survival time of the high RiskScore sample is significantly less than that of the low RiskScore sample, suggesting that the former has a worse prognosis (Figure 3C). timeROC was used to analyze the ROC of RiskScore. The predictive classification efficiency of 1, 3, and 5 years is shown in Figure 3D, which demonstrates that the model has a high AUC. The samples greater than zero after zscore (85 samples) were divided into the high-risk group (85 samples) and samples less than zero after zscore were divided into the low-risk group (97 samples). The difference between the survival curves of the high- and low-risk groups is shown in Figure 3E (*p* < 0.0001).

### Verifying the Robustness of the Risk Model

**FIGURE 4:**
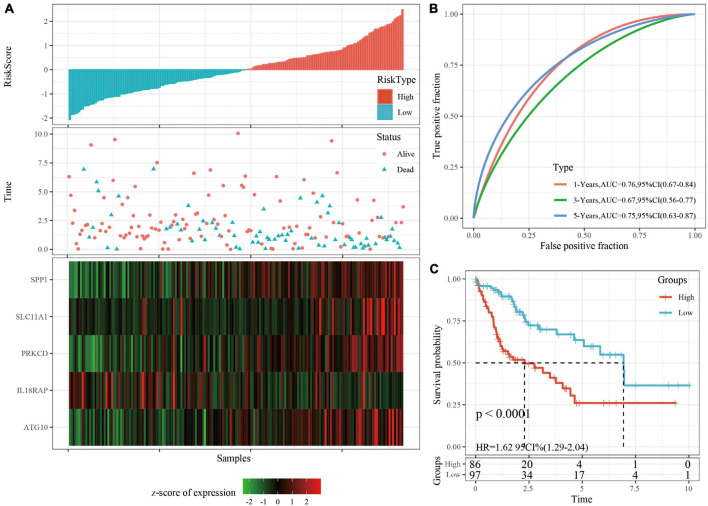
**(A)** RiskScore, survival time, survival status, and 5-gene expression in TCGA validation set. **(B)** ROC curve and AUC of 5-gene signature classification. **(C)** The KM survival curve distribution of 5-gene signature in validation set.

**FIGURE 5:**
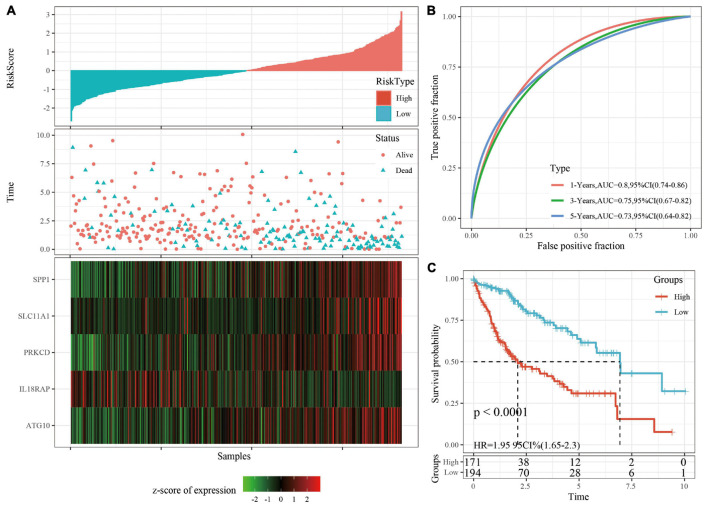
**(A)** RiskScore, survival time, survival status, and 5-gene expression in TCGA data sets. **(B)** ROC curve and AUC of 5-gene signature classification. **(C)** The KM survival curve distribution of 5-gene signature in TCGA data sets.

The robustness of the risk model was verified using internal data sets (TCGA validation set and all data sets) and external data sets (GSE14520 and HCCDB18), respectively. Using the same model and coefficients as the training set, the risk score of each sample was calculated according to the level of expression, and the RiskScore distribution of the sample was plotted. The RiskScore distributions of the TCGA validation set, all TCGA data sets, independent validation data sets GSE14520, and independent validation data sets HCCDB18 are shown in Figures 4A, 5A and Supplementary Figures 3A, 4A, respectively. The survival time of the high RiskScore sample is evidently less than that of the low RiskScore sample, indicating that the former has worse prognosis. The ROC curves of 1-year, 3-year, and 5-year predictive classification efficiency are shown in Figures 4B, 5B and Supplementary Figures 3B, 4B, respectively. The samples whose RiskScore was greater than zero after zscore were divided into the high-risk group and those with less than zero after zscore were divided into the low-risk group. In TCGA, 86 samples were divided into the high-risk group and 97 samples were divided into low-risk group. The survival curve indicated a difference between the two groups (*p* < 0.01) (Figure 4C). In all TCGA data sets, 171 samples were classified as high-risk and 194 were classified as low-risk. The survival curve indicated a difference between the two groups (*p* < 0.0001) (Figure 5C). In GSE14520, 110 samples were divided into the high-risk group and 111 samples were divided into the low-risk group. The survival curve indicated a difference between the two groups (*P* = 0.0084) (Supplementary Figure 3C). In the independent validation data set HCCDB18, 98 samples were divided into the high-risk group and 105 samples were divided into the low-risk group. The results indicated a difference between the two groups (*p* < 0.01) (Supplementary Figure 4C).

### Prognostic Analysis of Clinical Subgroups Based on RiskScore

**FIGURE 6:**
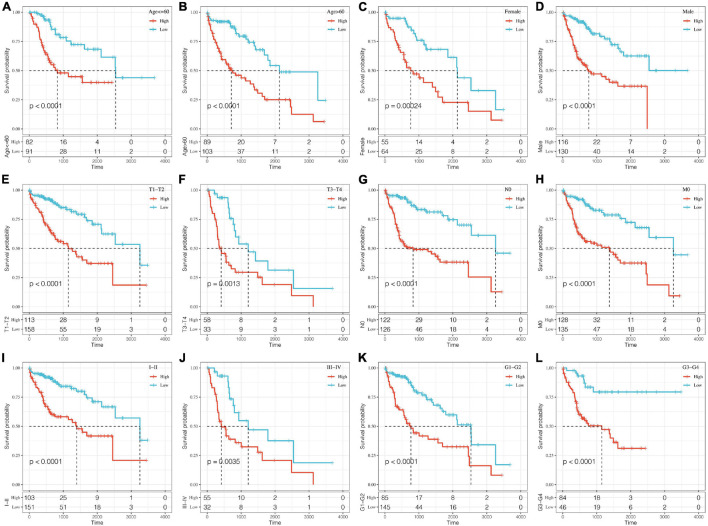
Prognostic analysis of clinical subgroups based on RiskScore. The horizontal axis represents survival time, and the vertical axis represents survival probability. Blue represents low expression group; red represents high expression group. **(A–L)** Based on Riskscore, survival curves were analyzed for Age ≤ 60, Age > 60, Female, Male, T1+T2, T3+T4, N0, M0, Stage I+II, Stage III+IV, Grade 1+2, and Grade 3+4 groups, respectively.

**FIGURE 7:**
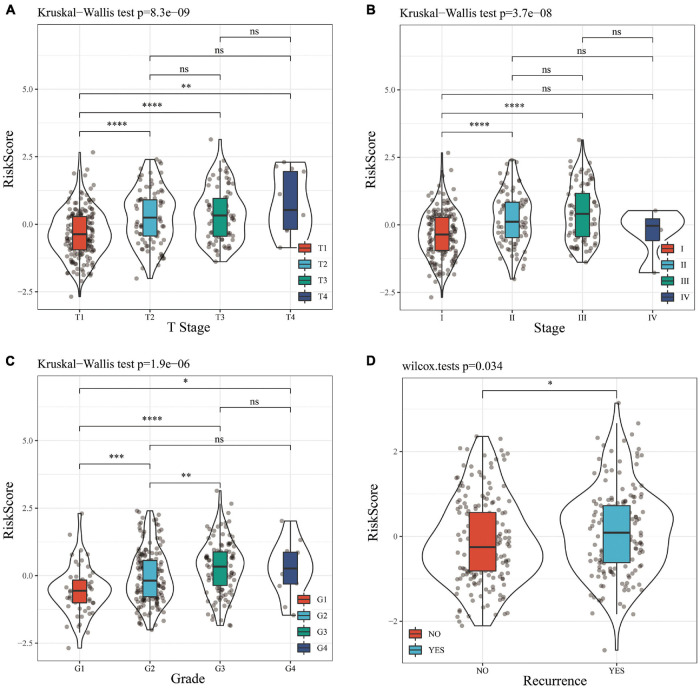
**(A)** Comparison of RiskScore among T Stage grouping samples. **(B)** Comparison of RiskScore among stage grouping samples. **(C)** Comparison of RiskScore among grade grouping samples. **(D)** Comparison of RiskScore between grouped samples with or without recurrence. * means *P* value < 0.05; ** means *P* value < 0.01; *** means *P* value < 0.005; **** means *P* value < 0.001; *ns*, no significant.

A stratified analysis of clinical subgroups variables including Age, Gender, TNM stage, Clinical Satge, and Grade were performed based on the expression of RiskScore. The results showed that RiskScore can classify Age ≤ 60, Age > 60, Female, Male, T1 + T2, N0, The M0, Stage I + II, Stage III + IV, Grade 1 + 2, Grade 3 + 4 groups into two groups with significant prognosis differences (Figures 6A–L). Therefore, our prognostic score can be used as a prognostic marker for clinical subgroups. Comparison of the distribution of RiskScore among TCGA clinical feature groups indicated that the RiskScore differed between T Stage, Stage, Grade, and relapse groups (Figures 7A–D, *P* < 0.05).

### The Expression of RiskScore on Different Clinical Features and the Construction of Nomogram

**FIGURE 8:**
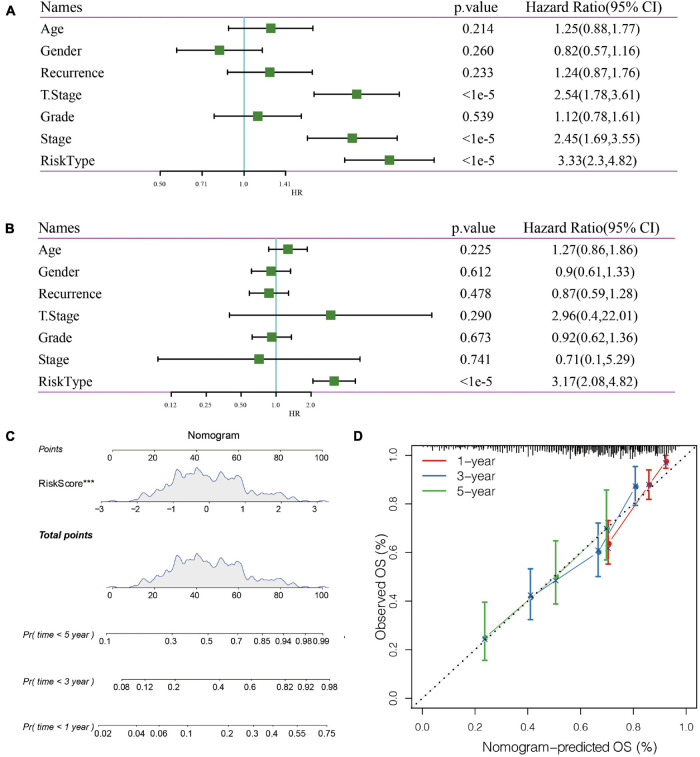
**(A)** Univariate Cox analysis. **(B)** Multivariate Cox analysis. **(C)** Nomogram model. **(D)**: 1-, 3-, and 5- year correction curve of the model.

In the TCGA data set, univariate and multivariate regression analysis showed a correlation between RiskScore and survival time (*p* < 0.05), indicating that 5-gene signature was independent in prognostic prediction (Figures 8A,B).

Nomogram can display risk model results directly and effectively. We constructed a nomogram according to the results of univariate and multivariate regression analysis (Figure 8C). Based on the results of RiskScore, as the RiskScore has the greatest influence on the survival prediction, the 5-gene risk model can predict the prognosis better. In addition, the accuracy of the 1-, 3-, and 5-year nomogram was corrected, which proved that the method has good performance.

### Comparison of Immune Cell Score Between High-Risk and Low-Risk Groups

**FIGURE 9:**
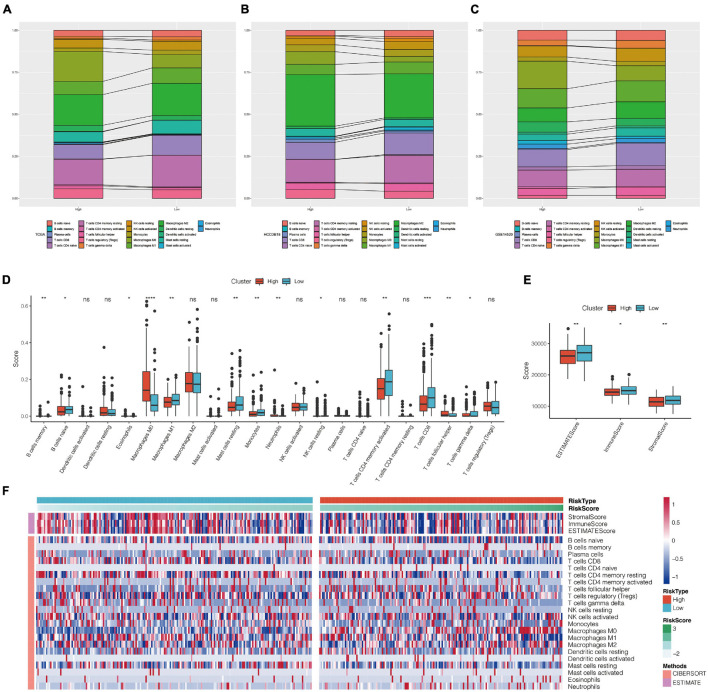
**(A–C)** Landscape of immune cell infiltration score in high-risk and low-risk groups of TCGA, HCCDB18, and GSE14520 data sets. **(D)** Comparison of CIBERSORT immune score between high-risk and low-risk groups in TCGA data set. **(E)** Comparison of estimate immune score between high-risk and low-risk groups in TCGA data set. **(F)** Heat map of correlation between CIBERSORT immune score and estimate immune score in high-risk and low-risk groups. *P < 0.05, **P < 0.01, ***P < 0.005, and ****P < 0.001; *ns*, no significant.

The distribution of 22 types of immune cells in TCGA, GSE14520, and HCCDB18 showed a consistent trend (Figures 9A–C). To visualize the differences, we analyzed the expression of StromalScore, ImmuneScore, ESTIMATE Score, and 22 types of immune cells in different RiskScore groups in the TCGA data set. Box plots showed that the high RiskScore group had significantly lower scores of Macrophages M1, Mast cell resting, and T cells CD8 than the low score group (Figure 9D). ESTIMATE, Immune Score, and Stromal Score had significantly higher immune scores in the low RiskScore group than the high RiskScore group (Figure 9E), suggesting that different expression characteristics of immune cells may promote prognosis in different populations. The association of CIBERSORT score and estimated scores of the high-risk and low-risk groups is shown in Figure 9F.

### Comparison of Risk Models

**FIGURE 10:**
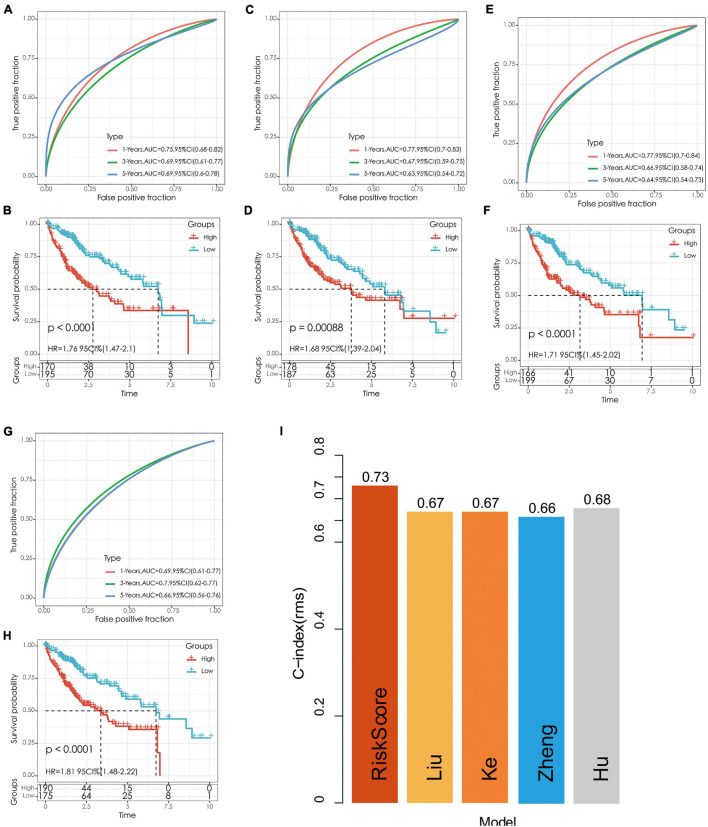
**(A,B)** The ROC curve of Hu’s risk model and the KM curve of high-risk and low-risk groups. **(C,D)** The ROC curve of Liu’s risk model and the KM curve of high-risk and low-risk groups. **(E,F)** The ROC curve of Ke’s risk model and the KM curve of high-risk and low-risk groups. **(G,H)** The ROC curve of Zheng’s risk model and the KM curve of high-risk and low-risk groups. **(I)** C-index comparison of different prognostic risk models.

We selected four prognostic risk models including Ke et al. (2018); Zheng et al. (2018), Liu et al. (2019), and Hu et al. (2020) to compare with our 5-gene model. In order to make the model comparable, we calculated the risk score of each OS sample in TCGA using the same method according to the corresponding genes in these four models. The samples greater than zero after zscore were divided into the high-risk group and those less than zero after zscore were divided into the low-risk group, after which the differences in OS outcomes between the two groups were calculated. The ROC and KM curves of the four models are shown in Figures 10A–H. Although OS prognosis differed between the high-risk and low-risk group samples among the four prognostic models, the AUC values at 1-, 3-, and 5-year are lower than those of our models. To compare the prediction performance of these models in LIHC samples, the rms package in R was used to calculate the concordance index (C-index) of the five models. The results showed that the C-index of our model was the highest (Figure 10I), indicating that the overall performance of our model is the best among the five models.

### Clinical Expression and Prognostic Value of the Five Genes in Pan-Cancer

**FIGURE 11:**
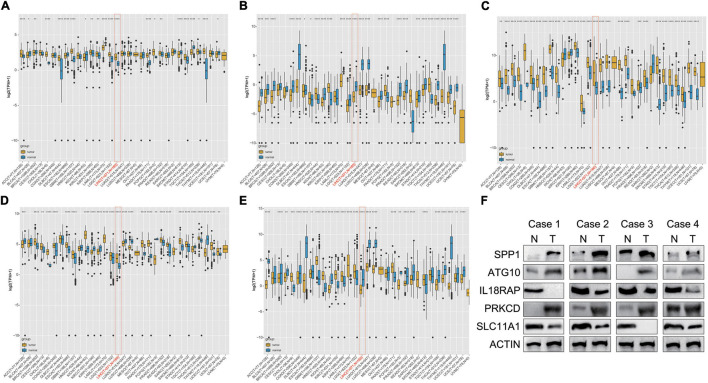
Differential box diagram of 5-gene expression in pan-cancer. **(A)** ATG10, **(B)** IL18RAP, **(C)** SPP1, **(D)** PRKCD, **(E)** SLC11A1, and **(F)** the protein expression of the 5 genes in 4 pairs of LIHC and normal samples. *P < 0.05, **P < 0.01, ***P < 0.005, and ****P < 0.001.

The results showed that mRNA levels of ATG10, IL18RAP, PRKCD, SLC11A1, and SPP1 differed between tumor tissues and normal tissues in most of the 33 cancer types. ATG10, SPP1, and PRKCD were overexpressed in HCC (Figures 11A,C,D), while IL18RAP and SLC11A1 had low expression (Figures 11B,E). Furthermore, IL18RAP had low expression in most tumors, while SPP1 was overexpressed.

We measured the protein expression of the 5 genes in 4 pairs of LIHC and normal samples. Compared with the normal samples, ATG10, PRKCD, and SPP1 were highly expressed in the tumor samples. Expression of IL18RAP and SLC11A1 were relatively higher in the normal samples than LIHC samples. The results were almost consistent with the database (Figure 11F).

**FIGURE 12:**
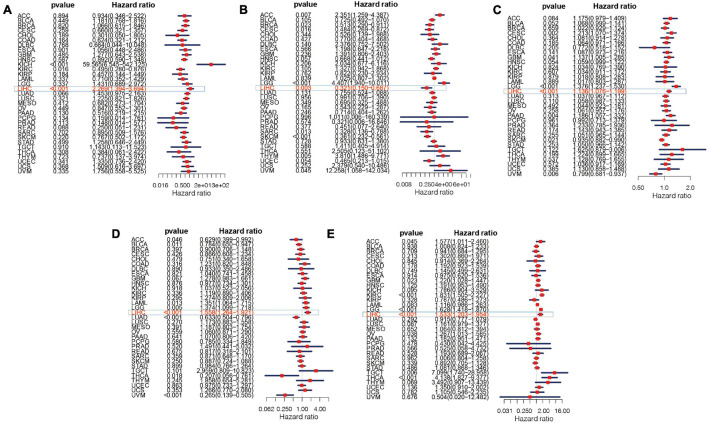
Survival curve of five genes in pan-cancer. **(A)** ATG10, **(B)** IL18RAP, **(C)** SPP1, **(D)** PRKCD, and **(E)** SLC11A1.

The overall survival analysis showed that ATG10, PRKCD, SPP1, and SLC11A1 were highly expressed in HCC with poor prognosis (Figures 12A,C,D,E). Besides, SPP1 was a high-risk gene in most tumors. The high expression of IL18RAP has a good prognosis in HCC and it is a protective gene in most tumors (Figure 12B).

### The Relation Between Five Genes and Tumor Microenvironment in Pan-Cancer

**FIGURE 13:**
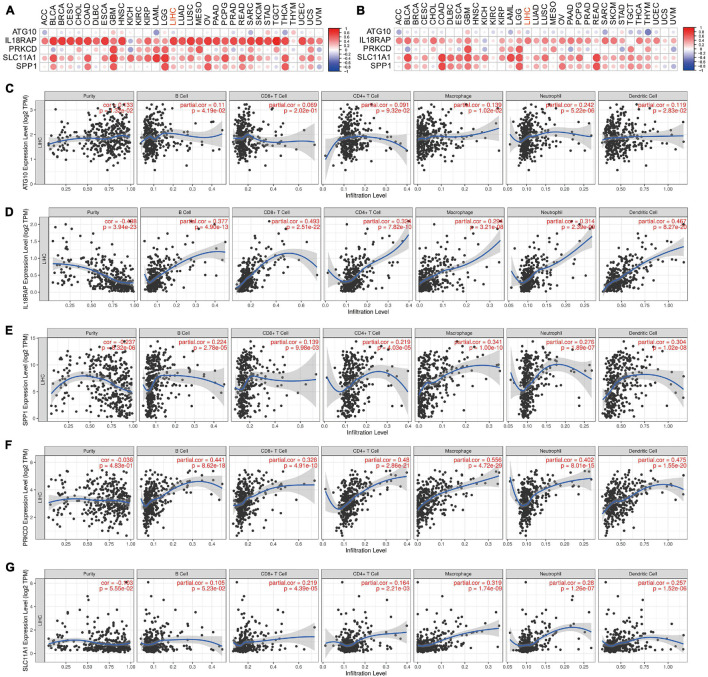
The correlation between five genes and tumor microenvironment. **(A)** The correlation between genes and immune core. **(B)** The correlation between genes and StromalScore. **(C–G)** The correlation between ATG10, IL18RAP, SPP1, PRKCD, SLC11A1, and immune cells.

The results showed that IL18RAP, PRKCD, SLC11A1, and SPP1 were positively correlated with the immune score in most tumors, including LIHC. This indicates that the immune score of most tumors increased with the increase of these genes’ expression. IL18RAP was strongly correlated with immune score in most tumors (Figure 13A). IL18RAP, SLC11A1, and SPP1 were positively correlated with StromalScore in most tumors, including LIHC. This indicates that StromalScore increased with the increase of IL18RAP, SLC11A1, SPP1 gene expression (Figure 13B). ATG10 was weakly correlated with immune core and StromalScore in tumors (Figure 13C). IL18RAP was highly correlated with B cell, macrophages, Neutrophil, dendritic cell, CD8 cell, and CD4 cell (Figure 13D). Both SPP1 and PRKCD were positively correlated with various types of immune cells, and were most strongly correlated with macrophages (Figures 13E,F). SLC11A1 also showed a high positive correlation with macrophages (Figure 13G).

### Potential Mechanism of Five Genes Involved in HCC

Studies of more than 10,000 tumor samples from 33 cancer types on the TCGA database have identified six immune subtypes: C1 (wound healing), C2 (INF-r dominant), C3 (inflammation), C4 (lymphocyte depletion), C5 (immunologically silent), and C6 (TGF-BETA predominates) (Thorsson et al., 2018). Since the C5 subtype is immunologically silent, we analyzed the association of the five genes with C1, C2, C3, C4, and C6 subtypes.

**FIGURE 14:**
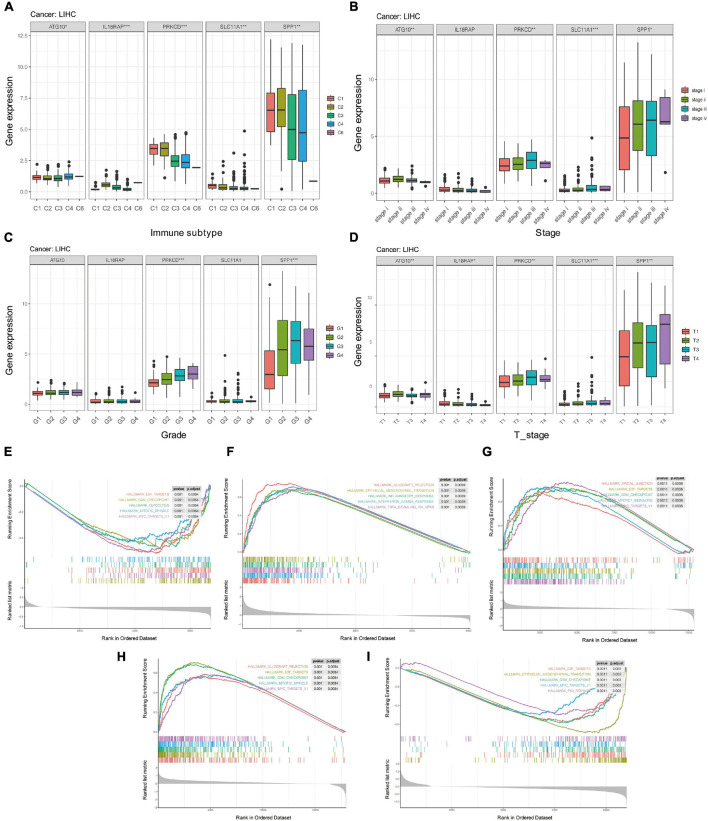
The potential mechanism of five genes in HCC. **(A)** Correlation between five genes and immune subtypes of pan-cancer. **(B)** Correlation between five genes and clinical stage. **(C)** Correlation between five genes and clinical grade. **(D)** Correlation between five genes and T stage. **(E–I)** GSEA enrichment analysis of ATG10, IL18RAP, SPP1, PRKCD, and SLC11A1. *P < 0.05, **P < 0.01, and ***P < 0.001.

We further analyzed whether there were significant differences in the expression of these five genes among different clinical characteristics, including immune subtypes, clinical stages, tumor grades, and T stages of HCC. The results showed that levels of ATG10, IL18RAP, PRKCD, SLC11A1, and SPP1 varied among different immune subtypes and T stages (Figures 14A,D). Levels of PRKCD and SPP1 also varied among different clinical stages and tumor grades (Figures 14B,C). Pathway enrichment analysis showed that ATG10 was mainly enriched in HALLM ARK_GLYCOLYSIS and G2M_CHECKPOINT. IL18RAP was mainly enriched in EPITHELIAL_MESENCHYMAL_TRAN SITION, TNFA_SIGNALING_VIA_NFKB pathway. SPP1 was mainly enriched in HALLMARK_E2F_TARGETS, MTORC1_ SIGNALING pathway. PRKCD was mainly enriched in HALLMARK_ALLOGRAFT_REJECTION, HALLMARK_MI TOTIC_SPINDLE pathway. SLC11A1 was mainly enriched in EPITHELIAL_MESENCHYMAL_TRANSITION, P53_PATH WAY (Figures 14E–I).

## Discussion

Hepatocellular carcinoma is the most common type of primary liver cancer and the sixth most common cancer worldwide (Desai et al., 2019). Considered the second leading cause of cancer deaths, it causes more than 700,000 deaths worldwide each year and thus poses a serious threat to human health (Halegoua-De Marzio and Hann, 2014). The liver is a quintessential immune tolerance organ due to its unique immune microenvironment. By blocking the immune escape of tumor cells and killing liver cancer cells, immunotherapy may be the most promising treatment for completely killing HCC cells. The response to immunotherapy usually depends on the interaction between tumor cells and their surrounding immune microenvironment. Comprehensive and systematic analysis of immune-related genes in HCC is therefore helpful for guiding immunotherapy and prognosis for HCC patients.

In this study, we first analyzed the differentially expressed immune genes of 730 immune-related genes in tumor and para-cancer samples using four data sets: GSE22058, GSE25097, GSE64041, and GSE36376. The results showed that 64 immune-related genes were involved in the development and progression of HCC through immune pathways such as NF–kappa B signaling pathway, TNF signaling pathway, and Cytokine-cytokine receptor interaction. The over-activation of NF-kappa B has been found to be closely related to the pathogenesis of HCC (Dai et al., 2017). PIGU (phosphatidylinositol glycan anchor biosynthesis class U) can decelerate the malignant progression of HCC by activating the NF-kappa B signaling pathway and promoting immune escape (Wei et al., 2020). TNF-α is an important component of the inflammatory microenvironment of HCC: it can promote the expression of B7-H1 in HCC cells induced by IFN-γ, thereby activating adaptive immune tolerance. Using RankAggreg analysis, we selected the top 15 most closely associated immune genes, including the chemokine families CXCL14, CXCL12, and CXCL2. CXCL14 is responsible for the recruitment and maturation of immune cells and promotes the movement of epithelial cells, which helps to establish immune surveillance in normal epithelium (Westrich et al., 2020). In addition, CXCL14 suppresses human papillomavirus-associated head and neck cancer through up-regulation of MHC-1 expression and antigen-specific CD8T cell response (Westrich et al., 2019). DNA methyltransferase 1 (DNMT1) can impair the homing ability of cytotoxic T cells to tumor cells by down-regulating CXCL12 (Li B. et al., 2018). High expression of CXCL2 enhances neutrophil recruitment and activation in an autocrine and/or paracrine manner (Li et al., 2016).

Lasso regression analysis and AIC analysis were used to further compress the target gene and construct five immune gene prognostic models: ATG10, IL18RAP, PRKCD, SLC11A1, and SPP1. To explore the potential mechanism of five genes in the malignant progression of HCC, we further analyzed the expression and prognosis value of five genes in pan-cancer. The results suggested that IL18RAP and SLC11A1 genes had low expression in HCC, while SLC11A1 with high expression had poor prognosis. IL18RAP is a member of the interleukin receptor family and facilitates IL-8-influenced signal transduction by producing IFN-γ (Cheung et al., 2005). In renal cell carcinoma, the high expression of IL18RAP suggests a poor prognosis (Yamada et al., 2018). The IL18RAP polymorphism may be associated with malignant progression of esophageal carcinoma (Zhu et al., 2016), suggesting that IL18RAP may have tissue specificity in different types of cancer. ATG10 is an autophagic E2-like enzyme which has been found to be overexpressed in colorectal cancer (Jo et al., 2012) and lung cancer (Xie et al., 2016). The overexpression of ATG10 is also associated with lymphatic invasion and lymph node metastasis in colorectal cancer (Jo et al., 2012). ATG10 promotes the proliferation and migration of lung and colon cancer cells (Xie et al., 2016; Jo et al., 2017). Functional variation of ATG10 rs10514231 may be associated with malignant progression of HCC (Shen and Lin, 2019). PRKCD is a member of serine and threonine-specific protein kinase C family. It is not only a tumor suppressor, but also promotes the cell cycle. PRKCD was inhibited in HCC, and activation of PRKCD could decrease the viability of HCC cells (Nambotin et al., 2011). SLC11A1 is a phagocyte membrane protein expressed in monocytes (Bauler et al., 2017). As a pro-inflammatory factor, SLC11A1 is closely related to the occurrence and development of many inflammatory diseases and susceptibility to infectious diseases (Braliou et al., 2019; Xu and Yang, 2020). SLC11A1 has been shown to be associated with esophageal cancer susceptibility in South African populations (Zaahl et al., 2005). SPP1, also known as osteopontin, acts as a cytokine to promote the expression of interferon and IL-12 (Wu et al., 2019). SPP1 is thought to be an oncogene in tumors, and the high expression of SPP1 in HCC suggests a poor prognosis (Chen et al., 2010). The polymorphism of SPP1 may be one of the genetic factors of HBV clearance and HCC (Shin et al., 2007). When identifying M1 macrophage-related modules related to the prognosis of thyroid cancer by WGCNA, it was found that the 4-gene signature including SLC11A1 and SPP1 could be used to predict the prognosis of patients with thyroid cancer (Zhuang et al., 2020). By analyzing the correlation between five genes and tumor microenvironment, we found that SPP1, PRKCD, and SLC11A1 were highly positively correlated with macrophages. The results further confirmed our conjecture that SLC11A1 and SPP1 may promote the malignant progression of HCC by regulating M1 macrophages to M2 macrophages. The results of enrichment analysis showed that both IL18RAP and SLC11A1 were closely related to EMT signal pathway, which suggested that IL18RAP and SLC11A1 might promote the metastasis of HCC by regulating HCC cells. ATG10 gene is closely related to glycolysis signal pathway, and PRKCD gene is enriched in graft rejection-related pathway. Our study provides a new direction for the study of the mechanism of these five genes involved in the malignant progression of HCC. M1 macrophages can exert their anti-tumor and immune-enhancing effects by secreting inflammatory factors, chemokines, effector molecules, and TNF- molecules. Under the action of Lmur4 and IL-13, M1 macrophages can be polarized into M2 macrophages to inhibit immune response. The high infiltration of M1 macrophages is usually related to the increase of tumor survival (Edin et al., 2012; Jackute et al., 2018).

The 5-gene signature is robust in both internal and external data sets. Further analysis indicates that the high infiltration of Mast cell resting is associated with increased survival time in lung adenocarcinoma (Wang et al., 2020). The higher the CD8 level of T cells, the better the prognosis of HCC patients (Gabrielson et al., 2016). All the above studies confirmed that the high-risk score group with low Macrophages M1, Mast cell resting, and T cells CD8 scores had poorer survival outcomes, which confirmed our analysis. Additionally, the changes in immune cells provided insights into the molecular mechanism of the 5-gene signature in HCC.

However, first of all, as our study is a retrospective study based on public databases, lacking clinical validation of the prognostic value of the signature.

Secondly, based on the results of pathway enrichment analysis, prospective verification of the underlying biological mechanism of core genes is required.

And the population ethnicities in the TCGA database are mainly confined to White people and Black people, and extrapolating our findings to other ethnic groups needs to be substantiated. Finally, we will also explore the possibility of containing more variables to further improve prognostic performance.

## Conclusion

In conclusion, this study systematically described the immune features of HCC based on 730 immune-related genes, identified the genes associated with prognosis, and constructed a 5-gene prognostic risk model. Thus, it can provide a good prognosis evaluation for the HCC samples. Our study further elucidates the relationship between immune microenvironment and prognosis of patients with HCC and provides new insights for immunotherapy of HCC.

## Data Availability Statement

The original contributions presented in the study are included in the article/Supplementary Material, further inquiries can be directed to the corresponding author.

## Ethics Statement

The patients and their families in this study were fully informed, and informed consent was obtained from the participants. This study was approved by the Ethics Committee of Hainan General Hospital.

## Author Contributions

DX, YW, and JW designed the study, performed data analysis, and wrote the manuscript. YZ, ZL, and YC performed data collection. JZ supervised the manuscript. All authors read and approved the manuscript.

## Conflict of Interest

The authors declare that the research was conducted in the absence of any commercial or financial relationships that could be construed as a potential conflict of interest.

## Publisher’s Note

All claims expressed in this article are solely those of the authors and do not necessarily represent those of their affiliated organizations, or those of the publisher, the editors and the reviewers. Any product that may be evaluated in this article, or claim that may be made by its manufacturer, is not guaranteed or endorsed by the publisher.

## Footnotes

1
http://lifeome.net/database/HCCDB/home.html

2
https://cistrome.shinyapps.io/timer/

## Supplementary Material

Supplementary Figure 1 **(A)** Venn diagram of differentially expressed immune genes. **(B–G)** The difference in expression of C6, EGR1, C8B, CFP, CXCL2, and CCL19 genes in tumor and paracancerous tissues, respectively. ***P* < 0.01 and *****P* < 0.0001.

Supplementary Figure 2 The KM curve of the five genes.

Supplementary Figure 3 **(A)** RiskScore, survival time, and 5-gene expression in GSE14520. **(B)** ROC curve and AUC of 5-gene signature classification. **(C)** KM survival curve distribution of 5-gene signature in independently validated data set GSE14520.

Supplementary Figure 4 **(A)** RiskScore, survival time, and 5-gene expression in HCCDB18. **(B)** ROC curve and AUC of 5-gene signature classification. **(C)** KM survival curve distribution of 5-gene signature distribution in HCCDB18.

## Abbreviations

AIC: the Akaike information criterion
DEGs: differentially expressed genes
DEIGs: differentially expressed immune genes
FDR: false discovery rate
GEO: gene expression omnibus
GO: gene ontology
GSEA: gene set enrichment analysis
HCC: hepatocellular carcinoma
KEGG: kyoto encyclopedia of genes and genomes
LIHC: liver hepatocellular carcinoma
TIIC: Tumor immune infiltration cell.
