# Supplementary figures and images for: Systematic Characterization of Novel Immune Gene Signatures Predicts Prognostic Factors in Hepatocellular Carcinoma
Source: Front Cell Dev Biol. 2021 Sep 23;9:686664. doi: 10.3389/fcell.2021.686664 (PMC8494981; doi:10.3389/fcell.2021.686664)

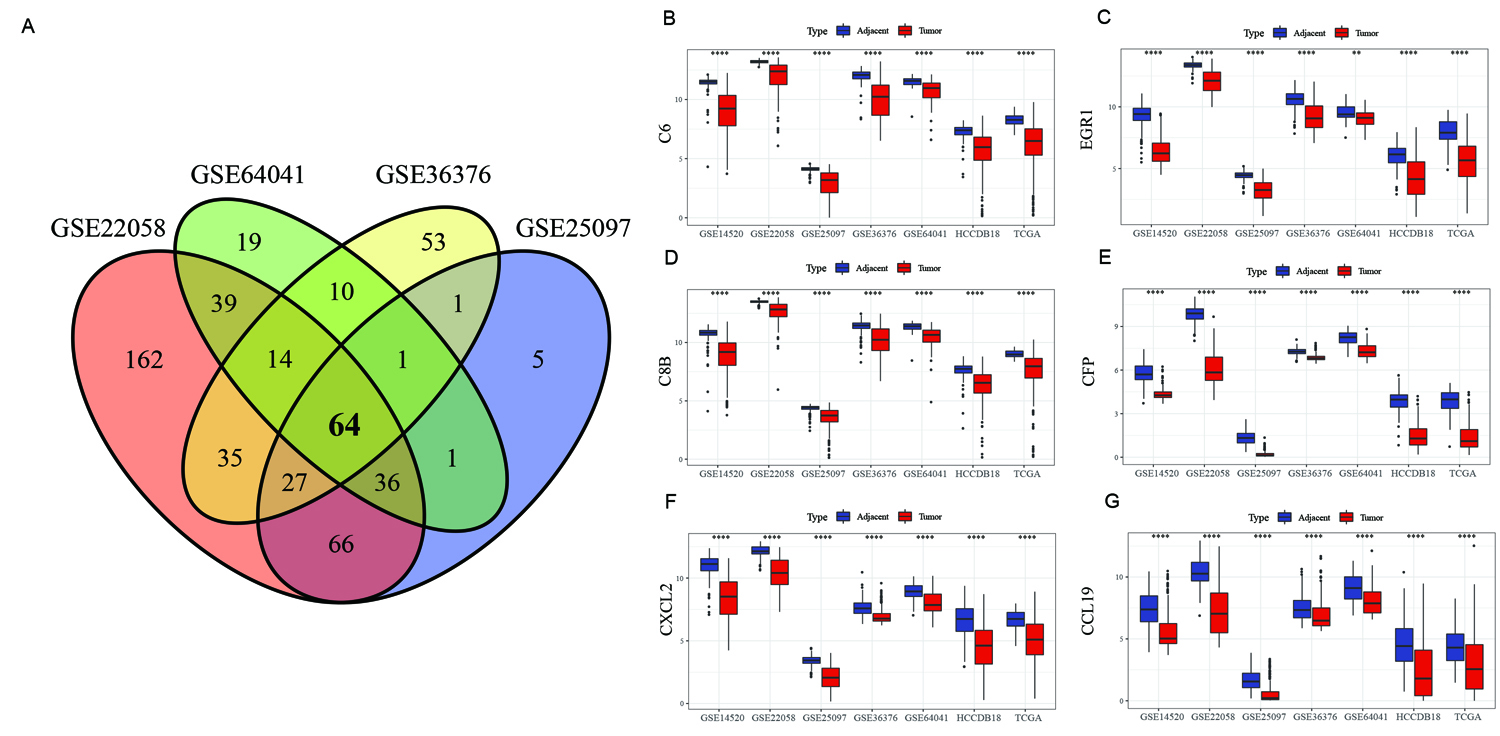

Supplement: Supplementary Figure 1 — (A) Venn diagram of differentially expressed immune genes. (B–G) The difference in expression of C6, EGR1, C8B, CFP, CXCL2, and CCL19 genes in tumor and paracancerous tissues, respectively. **P < 0.01 and ****P < 0.0001. [file Image_1.JPEG]

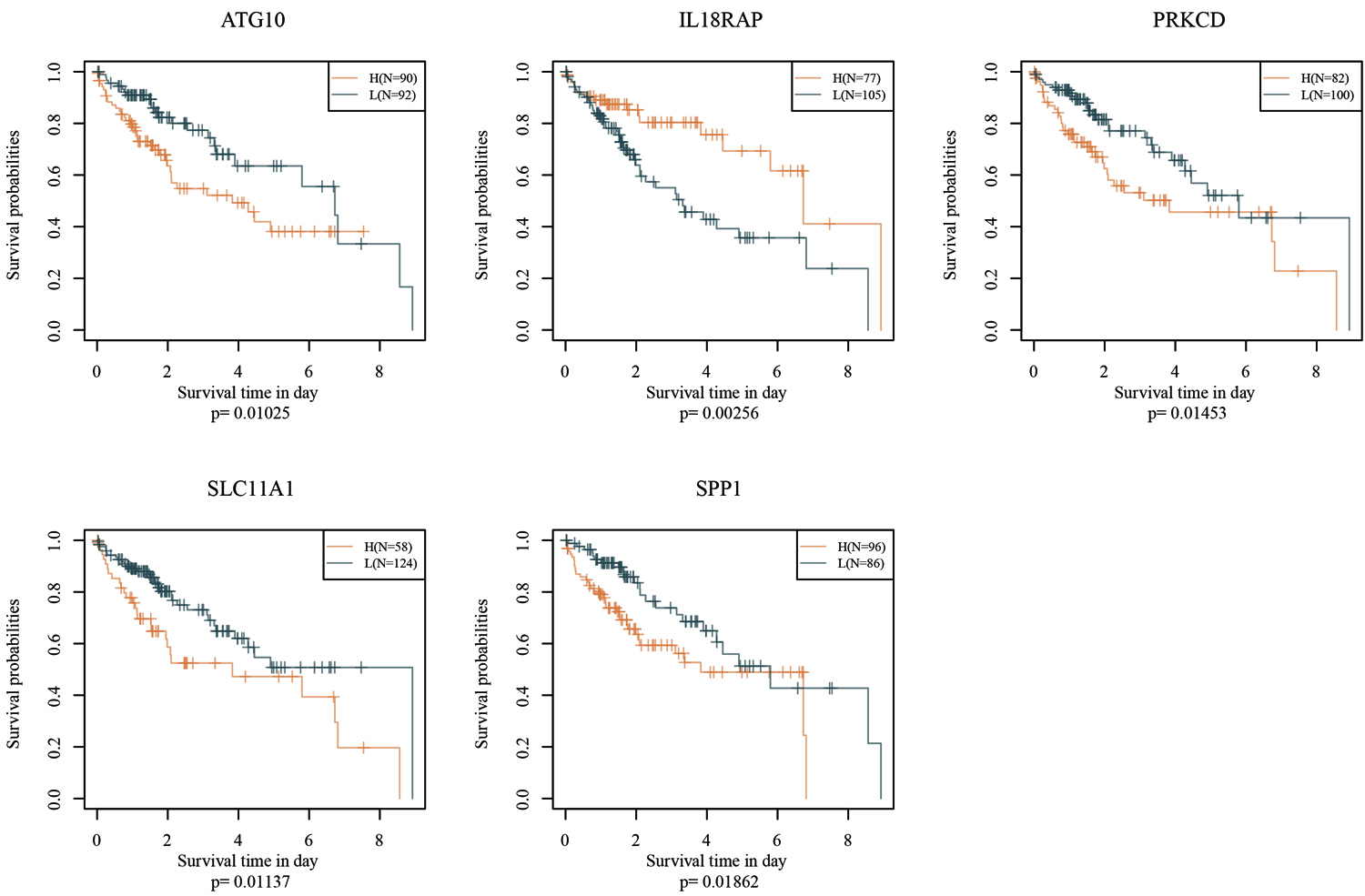

Supplement: Supplementary Figure 2 — The KM curve of the five genes. [file Image_2.JPEG]

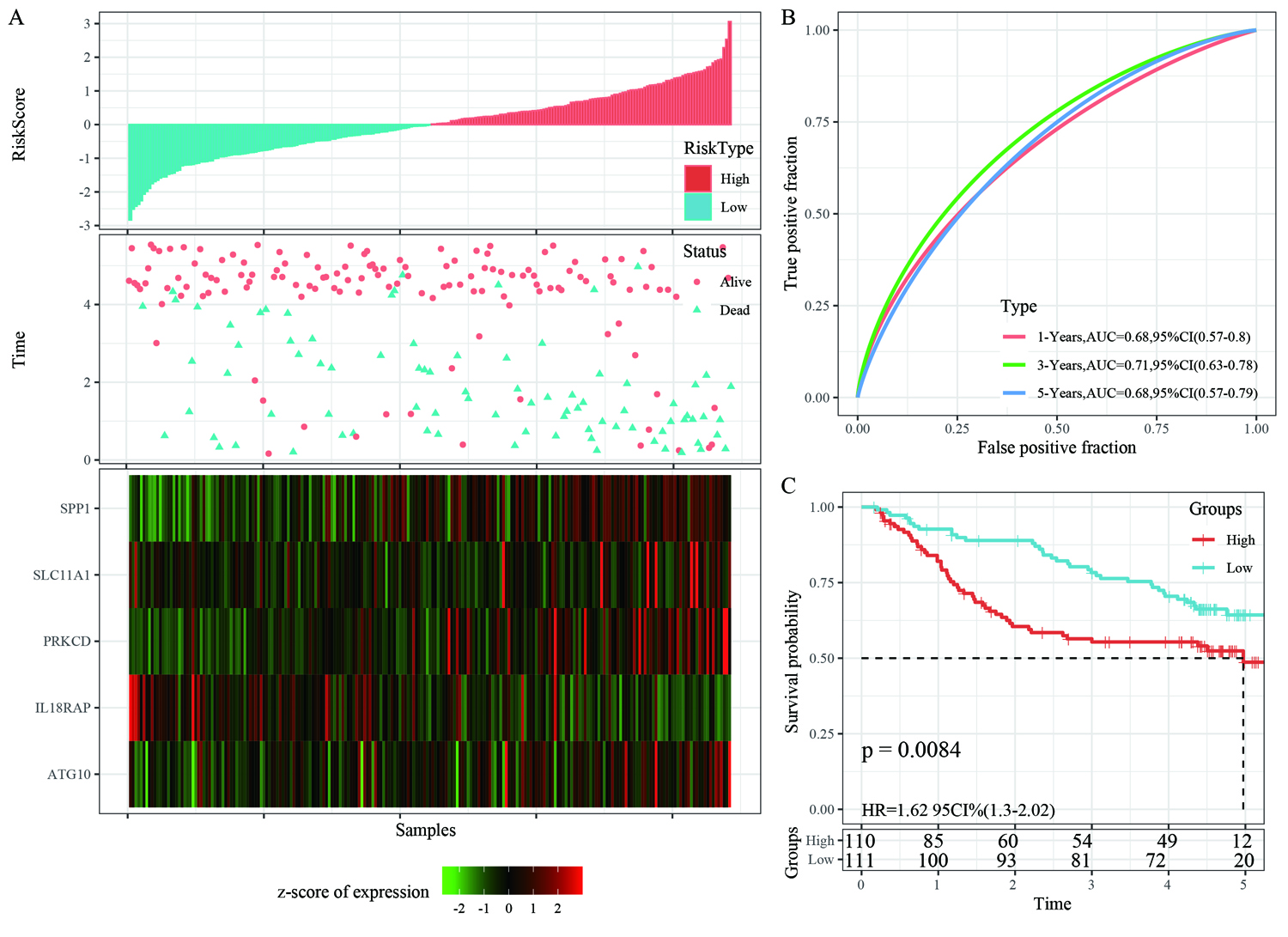

Supplement: Supplementary Figure 3 — (A) RiskScore, survival time, and 5-gene expression in GSE14520. (B) ROC curve and AUC of 5-gene signature classification. (C) KM survival curve distribution of 5-gene signature in independently validated data set GSE14520. [file Image_3.JPEG]

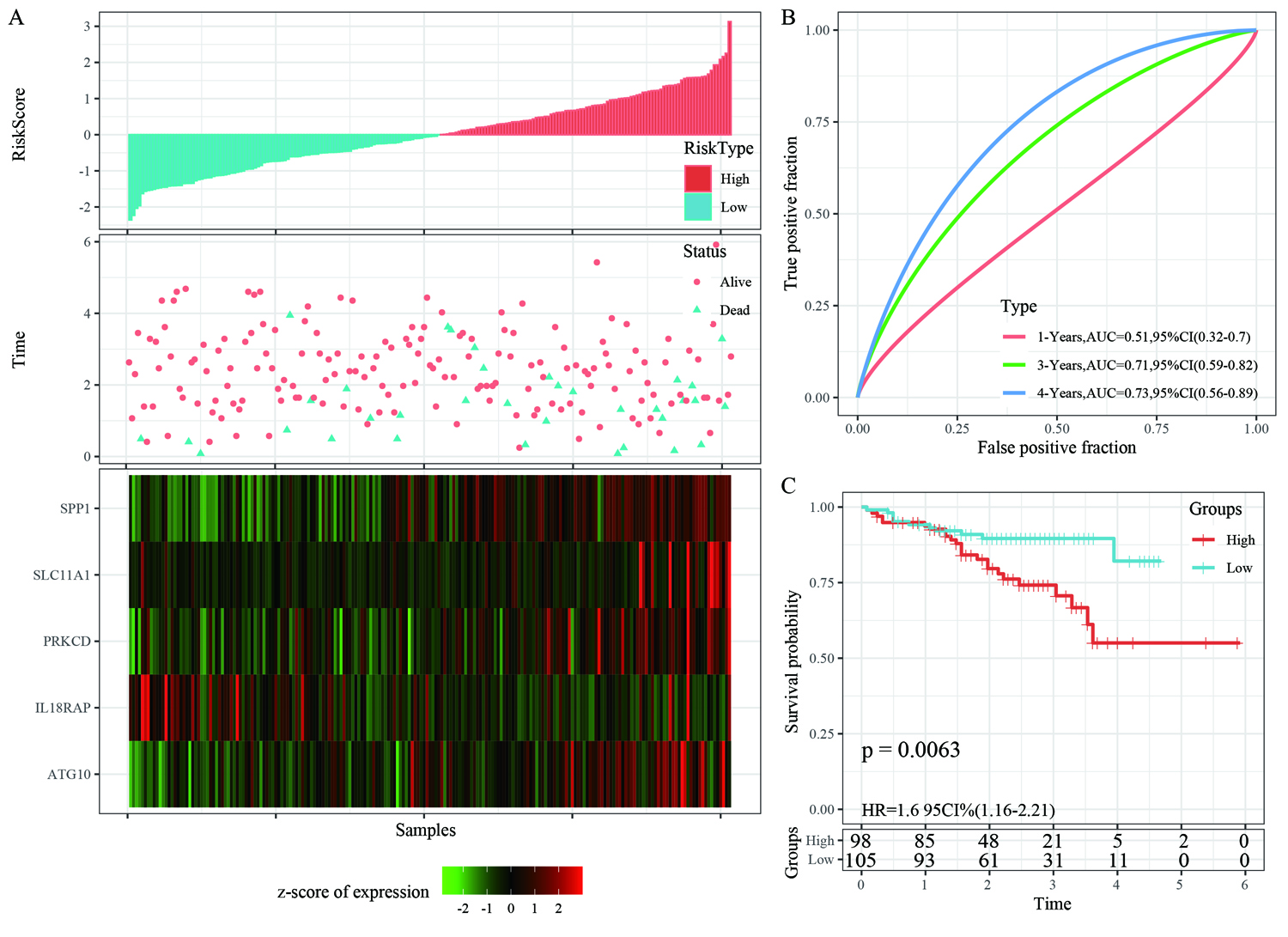

Supplement: Supplementary Figure 4 — (A) RiskScore, survival time, and 5-gene expression in HCCDB18. (B) ROC curve and AUC of 5-gene signature classification. (C) KM survival curve distribution of 5-gene signature distribution in HCCDB18. [file Image_4.JPEG]

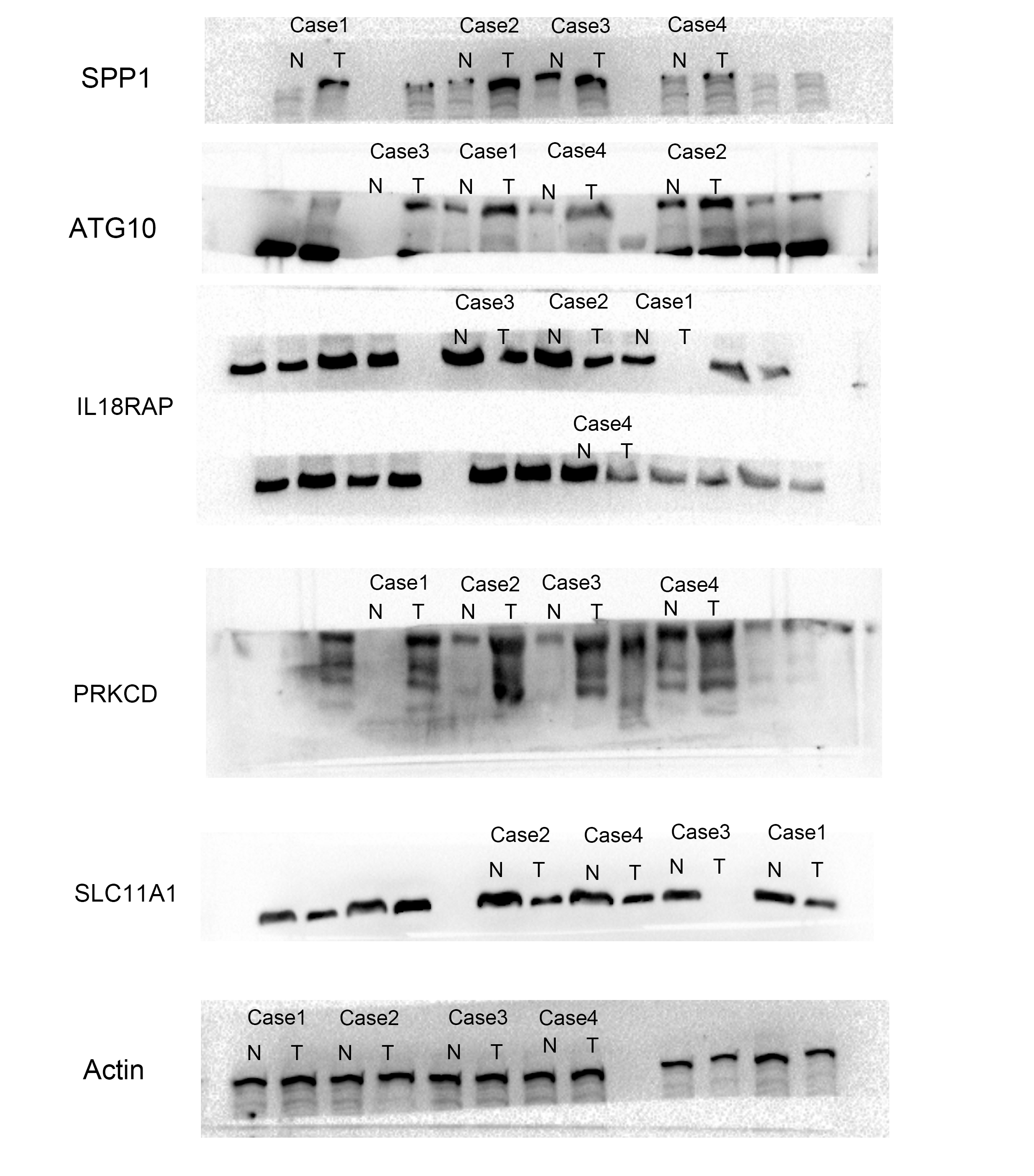

Supplement: Supplementary file 5 [file Data_Sheet_1.ZIP › Uncut full data.jpg]
